# Non-immunoglobulin E-mediated food allergy

**DOI:** 10.1186/s13223-024-00933-4

**Published:** 2024-12-19

**Authors:** Victoria E. Cook, Lori A. Connors, Timothy K. Vander Leek, Wade Watson

**Affiliations:** 1 Division of Allergy, Department of Pediatrics, University of British Columbia, Vancouver, BC Canada; 2 Department of Medicine, Dalhousie University, Halifax, NS Canada; 3 Department of Pediatrics, Faculty of Medicine and Dentistry, University of Alberta, Edmonton, AB Canada; 4 Division of Allergy, Department of Pediatrics, Dalhousie University, IWK Health Centre, Halifax, NS Canada

## Abstract

Non-immunoglobulin E (IgE)-mediated food allergies are characterized by delayed gastrointestinal (GI) manifestations that occur after exposure to an inciting food protein; they include food protein-induced allergic proctocolitis (FPIAP), food protein–induced enteropathy (FPE), and food protein-induced enterocolitis syndrome (FPIES). Although the exact mechanisms underlying these disorders are not well understood, non-IgE-mediated food allergies likely represent a spectrum of disease with shared pathophysiological processes. Typically, these non-IgE-mediated food allergies begin in infancy or early childhood, although FPIES can present across the lifespan, with increasing reports in adults in recent years. Diagnosing non-IgE-mediated food allergies can be challenging due to the lack of noninvasive confirmatory tests or biomarkers for most of these disorders and the non-specific nature of GI symptoms. Thus, the diagnosis is usually made clinically, and relies on a constellation of typical symptoms that improve upon removal of the culprit food. The primary approach to management of FPIAP, FPE and FPIES is avoidance of the triggering food, and a multidisciplinary management approach that includes allergy/immunology may be required to avoid unnecessary food restriction and guide food reintroduction. This review outlines the clinical manifestations, epidemiology, pathophysiology, diagnosis, management, and prognosis of these non-IgE-mediated food allergies.

**Supplementary Information:**

The online version contains supplementary material available at 10.1186/s13223-024-00933-4.

## Introduction

Food allergy is defined as an immune-mediated adverse reaction to food proteins and it is generally classified as immunoglobulin E (IgE)-mediated (see *IgE-mediated food allergy* article in this supplement), mixed IgE- and non-IgE-mediated (e.g., eosinophilic gastrointestinal disorders), and non-IgE-mediated [1–3]. The non-IgE-mediated food allergies that result in gastrointestinal (GI) symptoms are the focus of this review and include food protein–induced allergic proctocolitis (FPIAP), food protein–induced enteropathy (FPE), and food protein–induced enterocolitis syndrome (FPIES) (Tables 1, 2).

**Table 1:** Classification and diagnostic criteria for FPIAP, FPE and FPIES [4, 14]

FPIAP^a^	FPE^a^	Acute FPIES^b^	Chronic FPIES^b^
• Small amount of rectal bleeding in an otherwise healthy infant • Resolution of symptoms after relevant food antigens are removed from diet (maternal diet if exclusively breastfed) • Recurrence of symptoms upon reintroduction of culprit food(s) in diet • Exclusion of other cause of rectal bleeding	• Generally < 9 months of age at initial diagnosis but can present at older ages • Repeated exposure to causative food elicits GI symptoms without alternative cause, predominantly protracted diarrhea, vomiting and FTT • Histologic confirmation of the diagnosis by small bowel biopsy in a symptomatic child, showing villous injury, crypt hyperplasia and inflammation • Clinical and histological improvement after removal of offending food(s) • Exclusion of alternative causes	• Diagnosis requires that patient meet the major criterion plus ≥ 3 minor criteria **Major criterion**: • Vomiting 1–4 h after suspect food ingestion AND • Absence IgE-mediated allergic symptoms **Minor criterion**: • ≥ 2 episodes of repetitive vomiting with same food • Repetitive vomiting episode with a different food • Extreme lethargy • Marked pallor • Need for ED visit • Need for IV fluid support • Diarrhea within 24 h (usually 5–10 h) • Hypotension • Hypothermia	**Symptoms and severity**: Severe (when offending food is ingested on a regular basis): • Intermittent but progressive vomiting and diarrhea (occasionally with blood) • Possible dehydration and metabolic acidosis **Milder (lower doses with intermittent ingestion)**: • Intermittent vomiting and/or diarrhea • Poor weight gain/FTT • No dehydration or metabolic acidosis **Most important criteria for diagnosis**: • Resolution of symptoms within days after elimination of offending food(s) • Acute recurrence of symptoms (vomiting in 1–4 h, diarrhea in < 24 h, usually 5–10 h) when the food is reintroduced • Confirmatory OFC, or presumptive diagnosis if OFC not performed

*ED* emergency department, *FPIAP* food-protein-induced allergic proctocolitis, *FPE* food protein-induced enteropathy, *FPIES* food protein-induced enterocolitis syndrome, *FTT* failure to thrive, *GI* gastrointestinal, *IgE* immunoglobulin E, *IV* intravenous, *OFC* oral food challenge

^a^There are no definitive diagnostic criteria in the literature. These are the criteria generally used to diagnose FPIAP or FPE in clinical practice

^b^These criteria are likely to miss presentations in adults

**Table 2:** Comparison of key features of FPIAP, FPE and FPIES

	FPIAP	FPE	FPIES
Typical age of onset	Days of birth to 6 months of age (median ∼ 2 months)	First 9 months of life (typically within the first 1 to 2 months)	First weeks to months of life Can also occur in adults
Cardinal symptom(s)	Blood in stools	Protracted, non-bloody, diarrhea	*Acute:* vomiting 1–4 h after ingestion *Chronic:* intermittent but progressive vomiting and diarrhea In adults, abdominal pain and diarrhea more common than vomiting
Additional symptoms	Mucus in stools, diarrhea, painful flatus, anal excoriation	Vomiting, FTT	*Acute:* pallor, lethargy, hypotension, hypothermia *Chronic:* reflux, dehydration, poor weight gain, FTT
Implicated food proteins	*Breast-fed infants:* CM, soy, egg, corn and wheat, meat, fish, apple, carrot, nuts, and sesame *Formula-fed infants:* CM and soy	CM most common; soy, wheat and egg also implicated	CM, soy, grains, egg, meats, fish, fruits/vegetables, peanut and tree nuts
Work-up/Investigations	Lab/other investigations generally not required If diagnosis is unclear, consider: • FOBT • WBC Allergy testing not recommended Endoscopy with biopsy generally not indicated unless differential diagnoses are being considered	Lab investigations to assess for malabsorption Assessment of fecal AAT (marker of GI protein loss), where available Allergy testing not recommended Endoscopy with biopsy required to confirm diagnosis	Lab/other investigations generally not required Allergy testing generally not recommended
Laboratory abnormalities	Mild anemia, hypalbuminemia (rare), eosinophilia	Non-anion gap metabolic acidosis, hypoproteinemia, steatorrhea, sugar malabsorption, deficiency of vitamin K-dependent factors	Leukocytosis, neutrophilia, thrombocytosis, metabolic acidosis, methemoglobinemia
Endoscopy/histology	Mild, focal colitis, eosinophilic infiltration, lymphonodular hyperplasia	Villous atrophy, crypt hyperplasia, lymphocyte infiltration	Friable mucosa, ulceration, villous atrophy, crypt abscesses, inflammatory cell infiltration
Diagnosis	Clinical	Clinical + biopsy for histological confirmation	Clinical
Management	Avoidance of trigger food (from maternal diet if breast-feeding) Trial of extensively hydrolyzed or amino acid-based formula in either breastfed or formula-fed infants	BF infants: Avoidance of trigger food from maternal diet Formula-fed: extensively hydrolyzed formula (first-line); if not tolerated, use amino-acid formula	Avoidance of trigger food, supportive care with ondansetron, fluid rehydration (PO or IV), corticosteroids depending on severity
Natural history	Resolution of symptoms by age 1 year	Resolution of symptoms by age 2–3 years	*Infant onset:* resolution of symptoms by age 3–5 years *Adult-onset:* unknown although appears to persist in majority of adults

*AAT* alpha-1-antitrypsin, *CM* cow’s milk, *FOBT* fecal occult blood test, *FTT* failure to thrive, *IV* intravenous, *PO* by mouth, *WBC* white blood cell count

FPIAP and FPE most commonly present in infancy or early childhood, although there have been reports of onset at older ages [1, 2, 4–12]. FPIES can present throughout the lifespan, and there has been a rapid increase in reports of FPIES in adults in recent years [13–18]. Although the underlying pathophysiology of these non-IgE mediated food allergies is still poorly elucidated, shared pathophysiological mechanisms are suspected. FPIAP, FPE and FPIES likely represent a continuum of disease whereby the expression and severity of symptoms is dependent upon the affected segment of the GI tract [3]. For example, FPIAP symptomatology is induced by the localized inflammation of the distal colon, causing bloody stool in otherwise well-appearing infants. FPE predominantly affects the small intestine, resulting in lower digestive manifestations such as protracted diarrhea and malabsorption symptoms, potentially accompanied by failure to thrive. FPIES can affect the entire GI tract, predominantly causing symptoms of intractable emesis which can be severe enough to cause metabolic disturbances and hypovolemic shock [3]. There is also increasing evidence to suggest that these non-IgE-mediated food allergies may be related to atopic disease and epithelial barrier disorders, although this relationship has not yet been well-established [19].

Unlike IgE-mediated food allergy, the GI symptoms associated with non-IgE-mediated food allergies are typically delayed from hours to weeks after ingestion of the culprit food(s) [1]. Their diagnosis can be challenging given the lack of noninvasive confirmatory tests or biomarkers for most of these disorders and the fact that GI symptoms are non-specific, with some even seen in healthy infants. In general, the diagnosis is made clinically based on evidence of symptom improvement upon elimination of the culprit food from the diet and recurrence of symptoms on reintroduction. Therefore, it is important for physicians and other healthcare providers to be familiar with the key manifestations of these non-IgE-mediated food allergies and the common offending foods. This review focuses on the classification and presentation, pathophysiology, epidemiology, diagnosis, management, and prognosis of FPIAP, FPE and FPIES.

## Food protein-induced allergic proctocolitis (FPIAP)

### Classification and clinical presentation

FPIAP is typically considered a benign disorder affecting the distal colon that is characterized by blood and mucus in the stool (with or without diarrhea) in otherwise healthy, normally growing infants (Tables 1, 2; see also supplementary appendix). Onset of symptoms typically occurs within days of birth to 6 months of age (median ∼ 2 months) [2, 4, 6, 7, 9–12], although older presentations as late as 2–14 years of age have also been observed [8]. Symptoms resolve with restriction of the inciting food from the maternal or infant diet and recur upon reintroduction. Note that FPIAP was formerly referred to as cow’s milk (CM) protein allergy, however, it is now known that other foods (beyond CM) can trigger the disorder [20].

### Epidemiology

FPIAP is considered a common cause of rectal bleeding in infancy. Prevalence estimates vary widely from 0.16% to 17% in the general infant population, and 18–64% in infants with rectal bleeding [4, 9, 21–24]. The wide variation in estimates is likely due to variations in case definitions and methodologies used across studies, with less strict definitions associated with higher prevalence rates [25]. For example, a recent prospective study of healthy newborn infants by Martin et al. identified a cumulative incidence of 17% for FPIAP when diagnosed clinically by community pediatricians [23]. However, this study likely over-represented the prevalence as infants with occult blood in the stool were included and food reintroduction was not performed to confirm the diagnosis [25]. A study of infants with suspected FPIAP found that, after a period of elimination of the inciting food, 11% did not have symptoms upon reintroduction and were diagnosed with transient colitis rather than FPIAP [20]. These findings underscore the importance of a trial of food reintroduction to confirm the diagnosis of FPIAP and to justify ongoing dietary restrictions.

### Pathophysiology

Although the exact immunologic mechanisms responsible for FPIAP have not yet been fully elucidated, it is believed to result from antigen exposure occurring through maternal ingestion and transfer via breast milk or infant formula ingestion [2]. While endoscopy and biopsy are not typically conducted for the routine work-up and diagnosis of FPIAP, early studies identified eosinophilic inflammation in the rectosigmoid region in biopsy specimens of infants with FPIAP [2]. Moreover, some studies suggest the involvement of T cells in the pathophysiology of FPIAP [2, 19]. Recent research has implicated the gut microbiome and innate immune system in the pathophysiology of this non-IgE-mediated food allergy [19]. Liu et al. performed fecal microbiota transplantations in 19 FPIAP infants and found increased microbiota diversity and symptom resolution within 2 days of transplantation that was sustained at 15 months after transplantation, suggesting a potential role of altered gut microbiota in the development of FPIAP [26].

### Food triggers

The list of the most common causative foods for FPIAP varies based on the geographic location of studies and the frequently ingested foods in those locations [3]. In general, the most common causative foods in breast-fed infants with FPIAP include CM, soy, egg, corn, and wheat in the maternal diet, although other inciting foods such as meat, fish, apple, carrot, nuts and sesame have been described [6, 9, 20]. In formula-fed infants, FPIAP is generally caused by CM and soy [2]; extensively hydrolyzed formulas may elicit symptoms in up to 10% of cases [6, 20, 22]. Although most infants with FPIAP have a single food trigger, approximately 40% may have multiple food triggers [20, 22].

### Risk factors

Exclusive formula feeding and exclusive breastfeeding have been associated with an increased risk of FPIAP. In the prospective study by Martin et al. discussed above, infants fed a combination of both breast milk and formula during the first 4 months of life were 56% less likely than exclusively formula-fed infants, and 38% less likely than exclusively breastfed infants to develop FPIAP [23]. Exclusive formula feeding was associated with the greatest risk of developing FPIAP.

Studies have also found a family or personal history of atopy to be associated with FPIAP [22, 23, 27]. Interestingly, both prenatal exposure to calcium carbonate antacids (via maternal use) and infant use of acid suppression therapy for reflux (i.e., proton pump inhibitor or histamine H2-receptor antagonist medications) have recently been associated with an increased risk of developing FPIAP [23, 28].

### Diagnosis

**Fig. 1:**
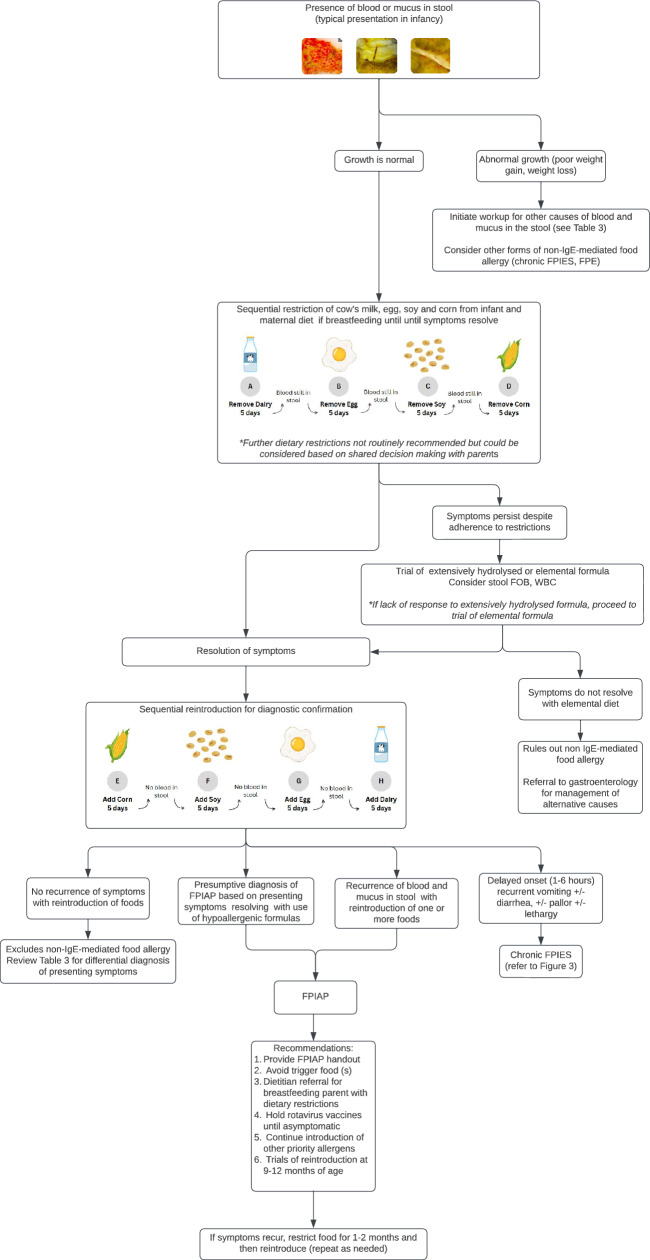
Algorithm for the diagnosis and management of FPIAP. Images in figure are taken from the FPIAP handout (see supplementary appendix). The complete handout is also available at https://www.allergyvic.com/qi-project. The FPIAP handout was generated by: Francesca Reinbolt, Delane Peters & Dr. Scott Cameron, and updated by Matt Griffin, Dr. Victoria Cook & Dr. Scott Cameron in October 2024. *FOB* fecal occult blood, *FPE* food protein-induced enteropathy, *FPIES* food protein-induced enterocolitis syndrome, *FPIAP* food-protein-induced allergic proctocolitis, *IgE* immunoglobulin E, *WBC* white blood cell count

The diagnosis of FPIAP relies on a detailed medical history, physical examination, and response to a trial of elimination of the suspected food(s) followed by reintroduction [1–4, 9–12, 29] (Table 1 and Fig. 1). Following removal of the culprit food, symptoms typically resolve in 1–2 weeks. Mucus in the stool may persist after blood in the stool has resolved. One study found that blood in the stool resolved after a median of 3 days (latest 2 weeks) while the resolution of mucus took longer (median 30 days) [20]. Return of symptoms upon reintroduction of the inciting food confirms the diagnosis of FPIAP.

The physical examination in infants with FPIAP is usually normal and infants generally appear healthy. Growth parameters should be assessed since growth is not typically impacted in infants with FPIAP.

Laboratory testing and other investigations are generally not necessary in well-appearing infants with typical symptoms of FPIAP. In cases where the diagnosis of FPIAP is uncertain, a fecal occult blood test (FOBT) and stool white blood cell (WBC) count may be considered, though both lack specificity. One study found that over one-third of healthy infants had false positive results on the FOBT [30].

Some studies have examined the diagnostic utility of fecal calprotectin (a neutrophil-derived protein that serves as a marker of intestinal inflammation), which has been shown to be elevated in infants with FPIAP and improve following food elimination [31]. However, it is a non-specific marker as fecal calprotectin levels have been found to be higher in infants compared to older individuals, and levels appear to decrease over time in healthy infants as well [32, 33]. Therefore, it is currently not recommended for the workup of infants with FPIAP.

Skin prick testing and serum food-specific IgE testing are not recommended for the diagnosis of FPIAP [9]. These tests evaluate for the presence of food-specific IgE and will be inconclusive in non-IgE-mediated GI disorders. Patch testing is also not recommended as there is little evidence supporting its utility in diagnosing FPIAP [4, 9]. Furthermore, endoscopy with biopsy is generally not indicated unless symptoms do not resolve with dietary elimination and alternative diagnoses are being considered [9].

### Differential diagnosis

The differential diagnosis of blood and mucus in the stool is broad and includes several allergic, GI, infectious and hematologic disorders, among others (Table 3) [3, 9, 34]. Key signs and symptoms that suggest a diagnosis other than FPIAP include an unwell appearance, fever, poor weight gain or weight loss, failure to thrive, severe diarrhea, vomiting and lack of symptom resolution upon elimination of the food trigger.

**Table 3:** Differential diagnosis of non-IgE-mediated food allergy

	FPIAP	FPE	Acute FPIES	Chronic FPIES
Allergic	FPIES FPE Eosinophilic gastroenteropathies	Celiac disease Chronic FPIES Eosinophilic gastroenteropathies	Anaphylaxis Eosinophilic gastroenteropathies	FPIAP FPE Eosinophilic gastroenteropathies
Infectious	Viral/bacterial/parasitic gastroenteritis	Viral/bacterial/parasitic gastroenteritis	Sepsis Viral/bacterial/parasitic gastroenteritis	Viral/bacterial/parasitic gastroenteritis
Gastrointestinal	Anal fissure Swallowed maternal blood NEC Intussusception Volvulus Meckel diverticulum Intestinal duplication cyst Infantile polyp VEOIBD	VEOIBD Cystic fibrosis	Hirschsprung Pyloric stenosis Intussusception Volvulus NEC	GERD Hirschsprung Pyloric stenosis VEOIBD Cystic fibrosis
Metabolic	–	Inborn errors of metabolism Congenital disaccharidase deficiency T1DM	Inborn errors of metabolism T1DM	Inborn errors of metabolism T1DM
Hematologic	Coagulation defect Thrombocytopenia	–	Congenital methemoglobinemia	Congenital methemoglobinemia
Neurologic	–	–	Cyclic vomiting Intracranial mass	Cyclic vomiting Intracranial mass
Cardiovascular	Vascular malformation	–	Congenital heart defect Cardiomyopathy Arrythmia	Congenital heart defect Cardiomyopathy
Endocrine	–	Congenital adrenal hypoplasia	Congenital adrenal hypoplasia	Congenital adrenal hypoplasia
Immunologic	–	IEI Autoimmune enteropathy	–	IEI Autoimmune enteropathy
Psychologic	–	Food aversion Neglect	Food aversion	Food aversion

Table adapted from Labrosse 2020 [3]

*FPE* food protein-induced enteropathy, *FPIAP* food protein-induced allergic proctocolitis, *FPIES* protein-induced enterocolitis syndrome, *GERD* gastroesophageal reflux disease, *IEI* inborn errors of immunity, *NEC* necrotizing enterocolitis, *T1DM* type 1 diabetes mellitus, *VEOIBD* very early-onset inflammatory bowel disease

### Management

Once the diagnosis of FPIAP is confirmed, management involves avoidance of the culprit food (Fig. 1, Table 2, see also supplementary appendix for FPIAP handout) [1–4, 9–12, 29]. For breastfed infants, this involves strict elimination of the culprit food from the breastfeeding parent’s diet. Parents and children on elimination diets should be referred to a registered dietitian to minimize any potential negative impact of food avoidance on nutrition. Particular attention should be given to the need for calcium supplementation in breastfeeding parents avoiding CM. Attempts at reintroducing the inciting food(s) can be initiated between 6 and 12 months of age either directly into the infant’s diet or into the breastfeeding parent’s diet depending on readiness for solid food introduction, breastfeeding status, and caregiver preference. If symptoms recur upon reintroduction, the culprit food should be avoided for an additional 1–2 months before again attempting reintroduction.

Use of the milk ladder (a home-based, stepwise approach to milk introduction starting from extensively heated forms and graduating to unheated forms) has been proposed to guide the reintroduction of CM in CM-allergic infants with FPIAP. While there is no evidence for its efficacy for this indication, it is not considered harmful and may have a positive impact on parent quality of life [9].

In mild cases of FPIAP, a ‘watch and wait’ approach for 1 month before an elimination diet is started may be considered to see whether spontaneous resolution of symptoms occurs [9]. The decision to take this approach should be based on parental preferences and the potential risk for mild anemia with prolonged rectal bleeding.

### Prognosis

The prognosis of FPIAP is excellent, with most infants achieving tolerance to the responsible food trigger by 12 months of age [2, 6, 7, 9, 22, 23]. One study found that tolerance developed at a later age (∼30 months) in infants with FPIAP with initial symptoms accompanied by diarrhea (defined as ≥ 3 stools per day or an increase in the frequency of routine defecation) [22]. Up to 20% of breastfed infants with FPIAP have spontaneous resolution of bleeding without changes to the maternal diet [6].

## Food protein-induced enteropathy (FPE)

### Classification and clinical presentation

**Fig. 2:**
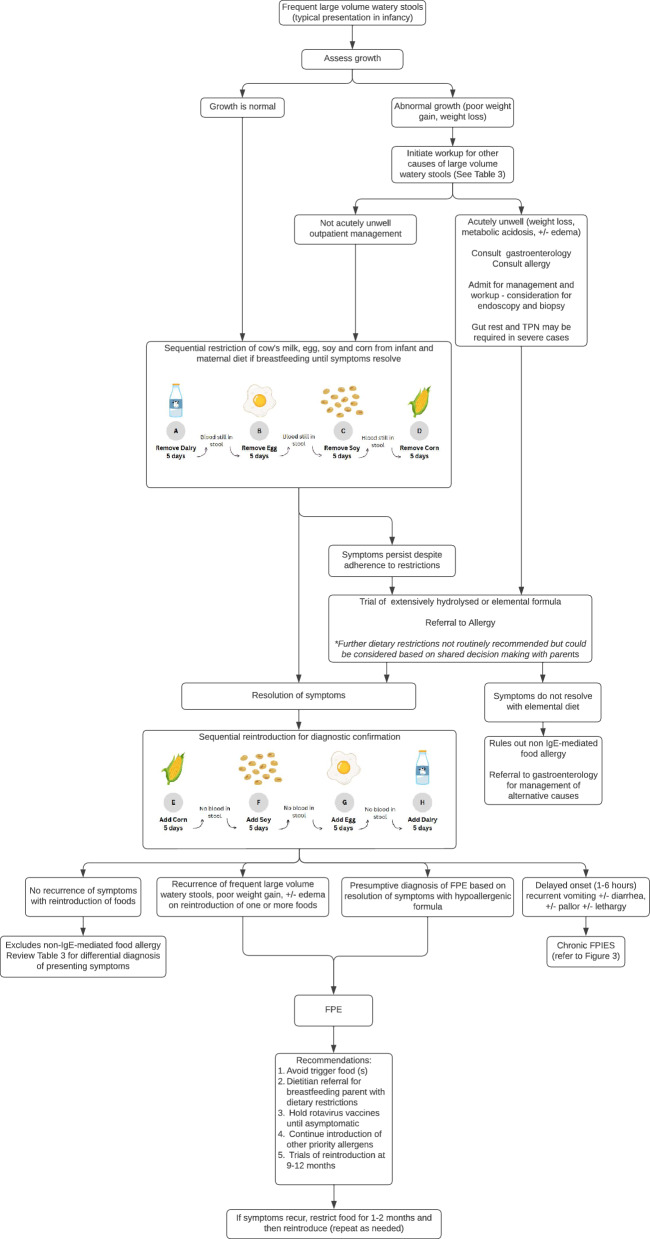
Algorithm for the diagnosis and management of FPE. Images in figure are taken from the FPIAP handout (see supplementary appendix). The complete handout is also available at https://www.allergyvic.com/qi-project. The FPIAP handout was generated by: Francesca Reinbolt, Delane Peters & Dr. Scott Cameron, and updated by Matt Griffin, Dr. Victoria Cook & Dr. Scott Cameron in October 2024. *FPE* food protein-induced enteropathy, *FPIES* food protein-induced enterocolitis syndrome, *IgE* immunoglobulin E, *TPN* total parenteral nutrition

FPE (formerly referred to as CM enteropathy) involves the small intestines and is characterized by protracted diarrhea with associated malabsorption and hypoproteinemia; it may also be associated with vomiting and failure to thrive, although bloody stools are usually absent [1, 4, 5] (Tables 1, 2; Fig. 2). Symptom onset is usually within the first 9 months of life (typically within the first 1 to 2 months), and often occurs within weeks after the introduction of CM formula [1, 4, 5]. Although there is one reported case from the 1960s of FPE being diagnosed in adulthood after presenting in later childhood [35], there have been no published reports of presentations in older children or adults in recent years. Like FPIAP, the symptoms of FPE resolve with removal of the causative food and recur on reintroduction.

### Epidemiology

Although the overall prevalence of FPE is unknown, it appears to be less common than FPIAP and FPIES, and reports suggest that the prevalence of this non-IgE-mediated food allergy has been declining [29]. However, a recently published case series of four infants with food-protein-induced protein-losing enteropathy suggests that FPE may be under-reported due to lack of clear diagnostic criteria for this condition [36].

### Pathophysiology

Eosinophils, CM-specific T-helper 2 lymphocytes, and localized production of IgE in the mucosa of the small intestine have been implicated in the pathophysiology of FPE [4]. Biopsy findings in patients with FPE have been well described and include atrophy of small intestinal mucosal villi, crypt hyperplasia, and lymphocyte infiltration [11].

### Food triggers

CM protein is the most common inciting food in FPE, although other food proteins, such as soy, wheat, and egg, have also been implicated [1, 4, 5].

### Risk factors

Little is known about possible risk factors for FPE. Earlier reviews on non-IgE-mediated food allergies indicated that FPE is associated with formula use as there have been no reports in exclusively breast-fed infants [2]. However, a recent case series observed the development of food-protein-induced protein-losing enteropathy in one infant that was exclusively breast-fed [36]. A personal history of atopy is estimated to be 22% in FPE, whereas family history of atopy is unknown [2].

### Diagnosis

A diagnosis of FPE should be suspected when the medical history reveals evidence of symptoms (e.g., protracted diarrhea, malabsorption, failure to thrive) on exposure to the food trigger, clearance of symptoms on removal of the trigger and recurrence upon reintroduction (Table 1; Fig. 2) [1, 2, 4, 29]. However, in severe cases where the infant requires hospital admission or parenteral nutrition, it may be prudent to forgo reintroduction of the food trigger and consider a presumptive diagnosis of FPE. The symptoms of FPE usually resolve within 1–4 weeks of elimination of the culprit food, although mucosal repair with normalization of disaccharidase activity may take several months [2, 4, 29].

During the physical examination, it is important to assess growth parameters as failure to thrive and inadequate weight gain are common in FPE. In addition, abdominal distension, and edema (due to protein loss) may also be observed [2, 4, 29].

Laboratory studies that assess for malabsorption of vitamins, minerals, proteins, and fats may be helpful in making the diagnosis of FPE. In cases of protracted diarrhea, patients may present with non-anion gap metabolic acidosis, hypoproteinemia, steatorrhea, sugar malabsorption, and deficiency of vitamin K–dependent factors. While blood in the stool is typically absent, occult blood can be found in 5% of patients [1]. Assessment of fecal alpha-1-antitrypsin (AAT) may also be considered in the work-up of patients with suspected FPE as elevated AAT in stools is a marker of GI protein loss [36], though this test is not widely available in Canada.

Similar to FPIAP, skin prick testing, serum food-specific IgE testing and patch testing are not recommended for the diagnosis of FPE [4, 9]. However, endoscopy and biopsy are necessary for the conclusive diagnosis of FPE; the diagnosis is confirmed by the presence of villous injury, crypt hyperplasia, and inflammation on small-bowel biopsy specimens [1, 2, 4].

### Differential diagnosis

Various allergic, infectious, GI, and immunologic disorders need to be considered in the differential diagnosis of FPE (Table 3) [3, 9]. As mentioned earlier, the characteristic signs and symptoms suggestive of FPE are persistent diarrhea and failure to thrive, which improve upon elimination of the inciting food trigger and recur upon re-exposure.

### Management

The management of FPE requires a multidisciplinary approach involving both gastroenterology and allergy/immunology (Fig. 2). Patients should be referred to a gastroenterologist, particularly in the acute period where admission may be necessary for severe cases requiring total parenteral nutrition and bowel rest. Referral to an allergist/immunologist is also imperative for ongoing management and guidance regarding eventual food reintroduction. With close clinical follow-up, trials of reintroduction of the food allergen can typically be considered at 9–12 months of age.

In breast-fed infants with FPE, strict avoidance of the offending food from the maternal diet is required. In formula-fed infants, an extensively hydrolyzed formula is the recommended first-line option [37]. If this is not tolerated or if the patient’s initial inciting trigger is extensively hydrolyzed formula, then an amino acid formula is required. Soy formula may be considered as an alternative option for those with CM allergy, although some experts recommend using it only in patients who are 6 months of age or older without evidence of failure to thrive given that soy may be a potential inciting allergen in FPE [37].

### Prognosis

Although the natural history of FPE is not as well described as FPIAP and FPIES, the prognosis is generally considered favorable with most infants achieving tolerance to the offending food protein by 2–3 years of age [29].

## Food protein-induced enterocolitis syndrome (FPIES)

### Classification and clinical presentation

Acute FPIES is characterized by profuse, repetitive vomiting that typically occurs 1–4 h (range: 30 min to 6 h) after ingestion of the trigger food (Tables 1, 2) [13, 14, 38]. Vomiting is often accompanied by lethargy, pallor, and diarrhea and, in rare severe cases, patients may experience hypotension, hypothermia, acidemia and methemoglobinemia. Symptoms may occur on first exposure to the trigger food or after a period of tolerance [13]. Like FPIAP and FPE, IgE-mediated skin and/or respiratory symptoms are typically absent in patients with FPIES.

Chronic FPIES occurs in the context of regular/ongoing ingestion of the trigger food. Presenting symptoms include intermittent vomiting, reflux, diarrhea and, in some cases, poor weight gain and failure to thrive (Table 1) [14, 39]. Symptoms resolve with elimination of the food trigger, but reintroduction induces an acute FPIES reaction (see “Diagnosis” for more details) [14].

Although FPIES typically presents in infancy, there have been increasing reports of FPIES in later childhood and adulthood, suggesting that onset of this non-IgE-mediated food allergy can occur throughout the lifespan [13–18]. The clinical presentation of FPIES in adults appears to vary somewhat from that in children, with abdominal pain and diarrhea being more common than vomiting in adult FPIES [15, 16, 18]. Case definitions and diagnostic criteria for FPIES are likely to be revised in the future to reflect the distinction in clinical presentation between adults and infants [17, 18].

### Epidemiology

Most epidemiologic data regarding FPIES has focused on its presentation in children [16]. Cumulative pediatric incidence rates estimated from population-based cohort or national survey studies in the United States (US), Israel, Australia, and Spain range from 0.14% to 0.7% [16, 40, 41]. Although the presentation of FPIES in adults has been less well-studied, a US population-based survey reported an estimated lifetime prevalence of 0.22% in adults 18 years of age and older [42].

Chronic FPIES appears to be less common than acute FPIES, accounting for 10–15% of FPIES cases in infants [15]. The prevalence of chronic FPIES in adults is unknown. To date, there has been only one reported case of chronic FPIES in an adult patient [15].

### Pathophysiology

The pathophysiology of FPIES remains incompletely understood but it is thought to involve innate immune responses and Th17-mediated signaling, as evidenced by increased eosinophils in blood and stool samples from patients with FPIES as well as eosinophil infiltration in intestinal biopsy specimens [19, 43, 44]. Although biopsies are not typically performed in patients with FPIES, those that have been examined also show villous blunting and increased monocytes with tumour necrosis factor (TNF)-alpha expression [44].

Neuroimmune pathways have also been implicated in FPIES, as suggested by reports of successful outcomes using the selective serotonin receptor antagonist ondansetron to ameliorate FPIES reactions [19, 43, 44]. Serotonin is known to play a pivotal role in GI functions, including motility, secretion, and vasodilation. Overproduction and secretion of serotonin by enterochromaffin cells in the GI tract can lead to activation of vagal afferents that initiate the vomiting reflex [43]. Serotonin has also been shown to attract eosinophils into the GI tract [19, 43, 44].

There is also some evidence suggesting that alterations in the gut microbiome may play a role in the pathogenesis of FPIES [19]. However, at present, there is insufficient data to draw any definitive conclusions about the role of these alterations in FPIES development.

### Food triggers

Evidence suggests that most patients with FPIES react to a single food trigger, and that multiple food triggers are less common [40]. The list of common food triggers for FPIES varies considerably based on the age of the population studied and the specific feeding/dietary practices in the countries where studies were conducted [40, 41]. In adult cohorts with FPIES, fish and shellfish, followed by egg, are the most reported triggers [15, 40, 41]. The most common trigger foods reported in North American studies of pediatric cohorts include CM, soy, grains and egg, although meats, fish, fruits/vegetables, peanut and tree nuts have also been reported as triggers [13]. Interestingly, there have been growing reports of peanut- and tree nut-triggered FPIES since the implementation of guidelines for early introduction of these foods in infants to prevent IgE-mediated food allergy (see *Primary prevention of food allergy: beyond early introduction* article in this supplement for more details on these guidelines) [45–47]. A dramatic increase in FPIES provoked by hen’s egg has also been observed in Japan following the publication of the country’s food allergy prevention guideline updates, which recommended early introduction of hen’s egg in high-risk infants [48].

An Australian study of 230 infants with FPIES found that reactions during exclusive breast-feeding are uncommon, occurring in only 5% of cases [38]. This study also found that that the majority of infants with CM-triggered FPIES were able to tolerate elemental formula (only 1 of 28 reacted). Approximately 21% (6 of 28) reacted to extensively hydrolyzed formula, 47% (8 of 17) reacted to soy formula, and 70% (7 of 10) reacted to partially hydrolyzed formula.

### Risk factors

An Australian population-based cohort study of infants with FPIES found that 7% had a sibling with FPIES and 57% had a family history of atopy [38]. A US population-based study found significantly higher rates of atopic disease in children and adults with FPIES compared to those without FPIES [42]. Approximately, 5% of families in this study reported having multiple children with FPIES.

Eosinophilic esophagitis (EoE) (see *EoE* article in this supplement) has also been reported to be associated with FPIES. In another US population-based cohort study, 19% of children and 13% of adults with EoE had a history of FPIES compared to 0.48% and 0.19% of children and adults, respectively, in the general population [49].

FPIES also appears to be associated with other GI pathologies. A 10-year prospective study found the prevalence of irritable bowel syndrome, EoE, inflammatory bowel disease and celiac disease to be higher in adults with FPIES (12.1%, 4.7%, 3.7% and 2.8%, respectively) compared to the general population (4.6%, 0.1%, 0.3% and 0.7%, respectively) [50]. Another study found high prevalence rates of FPIAP (23.2%) and gastrointestinal reflux disease (GERD) (36.0%) in patients with FPIES [51].

At present, it is unclear if sex may predispose patients to FPIES development. In the pediatric population, some studies have noted a slight predominance of FPIES in males, while others have found no considerable differences between males and females [15, 41]. In contrast, an overwhelming female predominance of FPIES in adults has been observed [16]. This finding should be interpreted with caution given the small numbers of adult patients in case cohorts reported to date.

### Diagnosis

**Fig. 3:**
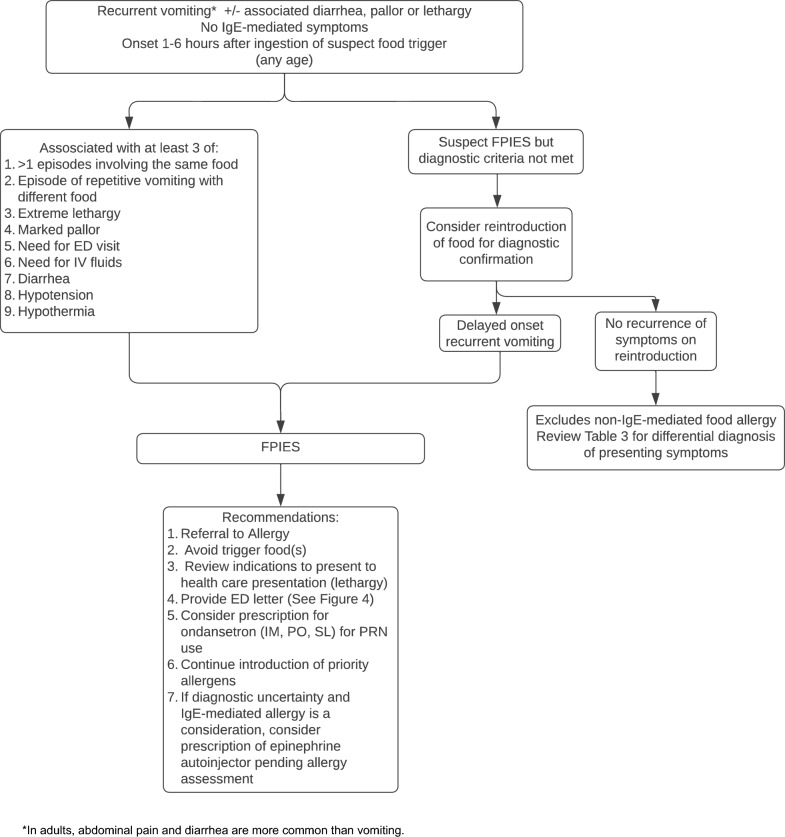
Algorithm for the diagnosis and management of FPIES. *FPIES* food protein-induced enterocolitis syndrome, *IgE* immunoglobulin E, *ED* emergency department,  *IM, intramuscular*, *IV* intravenous, *PO* oral, *SL* sublingual, *PRN* as needed

The diagnosis of FPIES is based on recognition of the typical clinical features of the disorder (Table 1; Fig. 3). During the physical examination, it is important to assess growth parameters since growth is generally normal in acute FPIES, but failure to thrive and inadequate weight gain are often observed in those with chronic FPIES [2, 4, 29]. Reintroduction of the trigger food is required to confirm a diagnosis of chronic FPIES. Resolution of symptoms within 3–10 days after the elimination of the offending food(s) followed by acute recurrence of symptoms upon reintroduction have been reported as the most important criteria for the diagnosis of chronic FPIES [14]. However, without a confirmatory challenge, the diagnosis of chronic FPIES remains presumptive.

Laboratory tests, endoscopy or other investigations are generally not indicated in the work-up of patients with suspected FPIES, but they may be helpful if alternative diagnoses are being considered. If blood work is performed acutely, infants with FPIES often show evidence of leukocytosis, neutrophilia and methemoglobinemia [2, 13].

Skin prick testing and serum food-specific IgE testing are not required for the diagnosis of FPIES. However, because food avoidance can increase the risk of IgE-mediated food allergy in susceptible individuals, these tests may be considered by the treating allergist prior to food reintroduction to gauge the patient’s risk of IgE-mediated allergy.

### Differential diagnosis

Several allergic, infectious, GI, and immunologic disorders need to be considered in the differential diagnosis of FPIES (Table 3) [3, 9, 18, 52]. Acute viral gastroenteritis is the most common diagnosis of exclusion for acute FPIES. Unlike FPIES, viral gastroenteritis is not associated with a specific food trigger and vomiting is often accompanied by diarrhea and fever [52]. Chronic FPIES with intermittent vomiting and/or diarrhea leading to failure to thrive has a broader differential diagnosis. In fact, FPIAP, FPE and EoE can present with similar symptoms to chronic FPIES. However, in these disorders, reintroduction of the food trigger is not associated with acute FPIES symptomatology [52].

### Management

In general, patients with FPIES should be referred to allergy/immunology for assessment, evaluation, and ongoing care (Fig. 3) [13, 14]. The standard of care for FPIES management is avoidance of the food trigger followed by reintroduction under specialist supervision (during a formal oral food challenge [OFC]) to see whether the disorder has resolved. Some practitioners may consider gradual home reintroduction in patients with prior mild reactions to large amounts of the trigger food (e.g., few episodes of vomiting, no or minimal lethargy) and who recovered at home without the need for healthcare intervention [52].

The likelihood of spontaneous resolution after a period of avoidance is lower in adults compared with children. Therefore, in adult patients, shared decision-making should be used to determine whether to proceed with reintroduction and OFC [16].

#### Treatment of acute episodes

The treatment of acute FPIES symptoms is supportive and tailored to the severity of symptoms [17, 18, 43, 53]. In the setting of mild reactions with no significant lethargy or signs of hypotension, enteral ondansetron and oral rehydration at home can be considered. A single dose of intravenous (IV) or intramuscular ondansetron (0.15 mg/kg; 2 mg for patients weighing 8–15 kg, 4 mg for those weighing 15–30 kg and 8 mg for those weighing > 30 kg) has also been shown to be effective for resolving vomiting and reducing the risk of dehydration in acute FPIES [13, 54].

In more severe cases with dehydration and hypotension, IV fluid boluses (10–20 mL/kg of normal saline) and IV corticosteroids (e.g., methylprednisolone 1 mg/kg to a maximum of 60–80 mg) are recommended [13, 17]. However, it should be noted that there are currently no studies demonstrating the efficacy of IV corticosteroids in the treatment of acute FPIES reactions.

#### At-home management (post-acute event)

As mentioned earlier, the primary management of FPIES consists of avoidance of the trigger food. Avoidance of other common FPIES triggers or allergenic foods during infancy is not recommended [13]. Current guidelines for the prevention of IgE-mediated food allergy (see *Primary prevention of food allergy: beyond early introduction* article in this supplement) emphasize the importance of early introduction of commonly allergenic foods at around 6 months of age (and not before 4 months), especially if the child is at risk for IgE-mediated allergy [55, 56]. Delays in the introduction of these foods increase the risk of developing IgE-mediated food allergy.

In contrast to IgE-mediated allergy, there is no need to avoid food products with precautionary (e.g., ‘may contain’) labelling and, in most cases, no need for maternal elimination of trigger foods in breastfed infants with FPIES [13].

Patients with CM- or egg-triggered FPIES who can tolerate extensively heated forms of these foods should be encouraged to continue consumption of these heated forms [17, 57]. In fish/shellfish-FPIES, there is evidence suggesting that some patients can tolerate alternate types of fish/shellfish other than the offending one [58, 59].

**Fig. 4:**
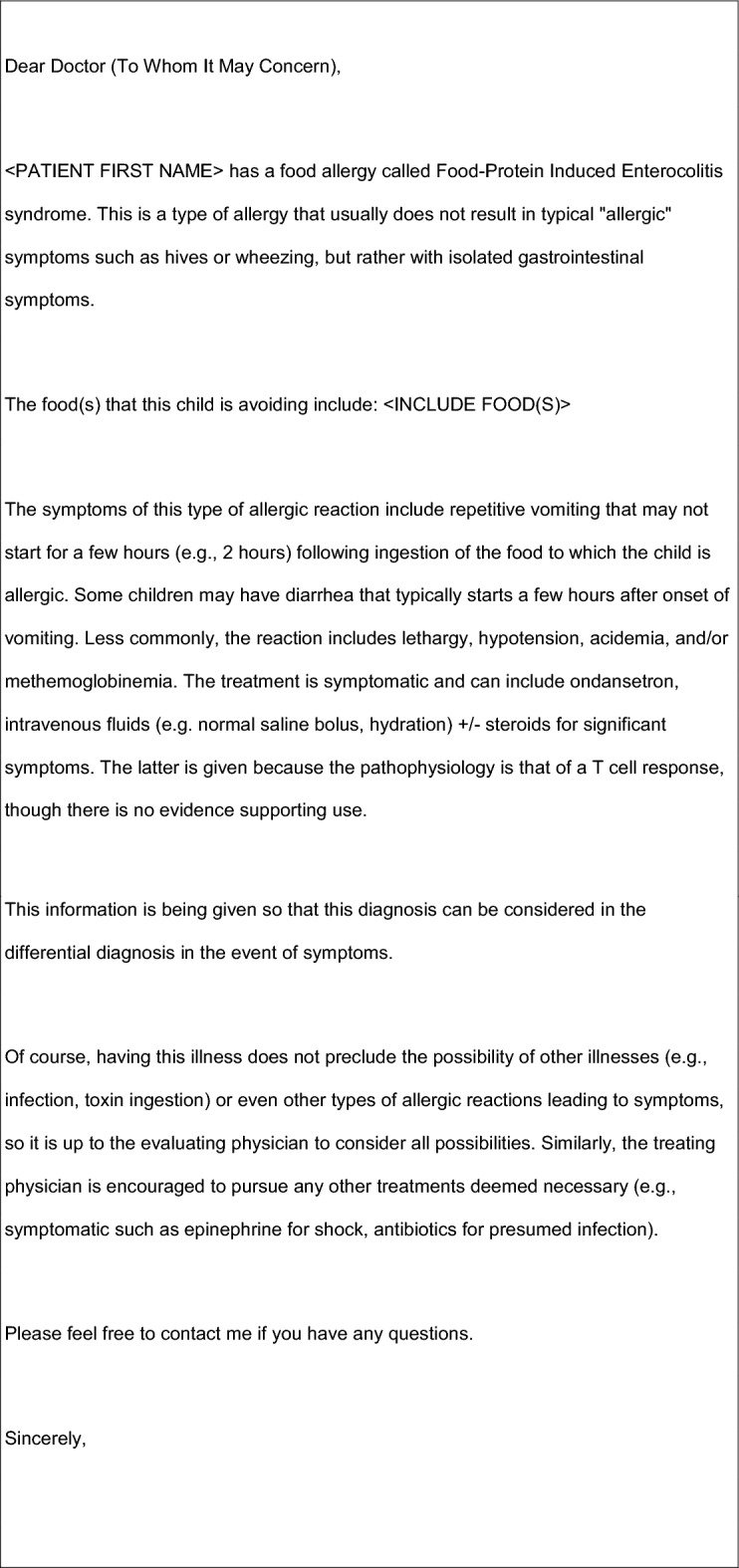
Example of pediatric ED letter. Letter adapted by Dr. Victoria Cook based on ED letter (for children) available on the International FPIES Association website at https://fpies.org/ (see “Resources” section)

Patients and families should be counseled on the signs and symptoms of FPIES reactions and, in the event of an accidental exposure, how to monitor for signs and symptoms of dehydration and hypotension that should prompt emergency evaluation [43]. Patients/parents should also be provided with a letter to bring to the emergency department (ED) that explains what FPIES is and provides recommendations for treatment [53]. An example of an ED letter is provided in Fig. 4.

Patients/families may also be provided with a prescription for ondansetron to be used as needed in the case of intractable vomiting upon accidental exposure to the food trigger [43, 53, 60]. Ondansetron has been associated with QT prolongation, so caution is recommended in patients with long-QT syndrome or other cardiac disease, or in those using other QT-prolonging medications [43, 53, 60].

An epinephrine autoinjector is not indicated for the treatment of FPIES as it has no effect on vomiting or other FPIES symptoms [13]. However, in cases where the diagnosis is unclear and there is suspicion of IgE-mediated food allergy, an epinephrine autoinjector could be prescribed until the patient is evaluated by an allergist. Epinephrine will not be harmful during an FPIES reaction.

### Prognosis

The prognosis of pediatric-onset FPIES is good, with most cases resolving by school age. A large retrospective review of over 400 children with FPIES found resolution rates of 35% by age 2 years, 70% by age 3 years, and 85% by age 5 years [61]. Evidence suggests that resolution rates and median age of resolution vary by type of FPIES trigger. CM-triggered FPIES has been reported to resolve earlier than FPIES triggered by fish or egg [40]. This is likely related to timing of food introduction as CM is generally introduced at an earlier age than other foods and, thereby, tolerance may be acquired earlier. Patients with fish-FPIES appear to have the lowest rates of resolution and the highest median ages of resolution. In egg-FPIES, evidence suggests that tolerance to cooked egg is achieved earlier than to raw egg [40].

There is limited data on resolution rates in adult-onset FPIES. In a prospective study that followed 107 adults with FPIES for a median of 6.2 years, only 16.8% achieved tolerance to the implicated food and ate it on a regular basis [50]. Ten patients achieved tolerance to small amounts of the triggering food. In those that achieved tolerance, the median duration was 3.5 years. This study also found that the longer adult patients had FPIES, the less likely it was to resolve (although age of FPIES onset did not correlate with time to resolution), and that fish- and crustacean-triggered FPIES was more likely to resolve than FPIES triggered by other foods [50].

## Conclusions

The non-IgE-mediated food allergies characterized by delayed onset of GI manifestations following exposure to an inciting food protein include FPIAP, FPE and FPIES. Although the underlying pathophysiology of these disorders is not well understood, they likely comprise a spectrum of disease with shared pathophysiological mechanisms. While onset of these non-IgE-mediated food allergies is typically in infancy or early childhood, there is emerging evidence to suggest that FPIES can present across the lifespan, with increasing reports in adults in recent years.

Management of these disorders relies on avoidance of the triggering food(s), and often requires a multidisciplinary approach. FPIAP can usually be diagnosed and managed by pediatricians and primary-care providers, although complex presentations may require referral to an allergist. FPE typically requires subspecialty management involving gastroenterology for acute care and allergy/immunology to guide eventual reintroduction of triggering foods. For FPIES, referral to allergy/immunology for OFCs and guidance on food reintroduction is imperative. Overall, the natural history of FPIAP, FPE and FPIES is largely positive, and most patients will be able to reincorporate triggering foods into their diet.

Research in the field of non-IgE-mediated food allergies is expanding, and expert opinions regarding diagnostic and management approaches are rapidly evolving. For FPIES in particular, current research is focused on interventions, such as earlier reintroduction of trigger foods to minimize the risk of developing IgE-mediated food allergy associated with prolonged avoidance and to reduce the negative impact of dietary restrictions on quality of life [62]. Other investigators are examining ways to reduce the need for costly, time-intensive food challenges in people with FPIES [57]. Diagnostic criteria are likely to be updated and revised in the future to better identify adults with FPIES [16, 17]. Future studies are needed to better characterize the pathophysiological mechanisms underlying non-IgE-mediated food allergies, identify potential biomarkers for improved diagnosis, and to optimize management practices.

## Key take-home messages

- Non-IgE-mediated food allergies include FPIAP, FPE and FPIES; they are characterized by delayed onset of GI symptoms, and likely have a shared underlying physiological process.
- While these disorders most commonly present in infancy and childhood, there is increasing recognition of adult-onset disease, particularly for FPIES.
- The diagnosis of non-IgE-mediated food allergies is based primarily on clinical history, with re-exposure to the food, and possibly food challenges, required for diagnostic confirmation in many cases.
- Management involves avoidance of trigger foods, with support to prevent nutritional deficiencies and mitigate the negative impact of food restriction.
- The natural history of these disorders is positive, with spontaneous resolution in most cases.
- Current research is focused on identification of biomarkers, improved characterization of presentations in adult populations, and management strategies that minimize the duration of food avoidance.

## Supplementary Information

Supplementary Material 1: Appendix: FPIAP Handout. This handout is available at https://www.allergyvic.com/qi-project. Permission for use of this handout was provided by Dr. Scott Cameron and Dr. Victoria Cook.

## Data Availability

Not applicable.

## Abbreviations

AAT: Alpha-1-antitrypsin
CM: Cow’s milk
ED: Emergency department
FOBT: Fecal occult blood test (FOBT)
FPE: Food protein-induced enteropathy
FPIES: Food protein-induced enterocolitis syndrome
FPIAP: Food-protein-induced allergic proctocolitis
FTT: Failure to thrive
GI: Gastrointestinal
IgE: Immunoglobulin E
IV: Intravenous
OFC: Oral food challenge
PO: By mouth
WBC: White blood cell count
