# Supplementary material for: Non-immunoglobulin E-mediated food allergy
Source: Allergy Asthma Clin Immunol. 2024 Dec 19;20(Suppl 3):70. doi: 10.1186/s13223-024-00933-4 (PMC11656650; doi:10.1186/s13223-024-00933-4)
Supplement: Supplementary file 1 — Supplementary Material 1: Appendix: FPIAP Handout. This handout is available at https://www.allergyvic.com/qi-project. Permission for use of this handout was provided by Dr. Scott Cameron and Dr. Victoria Cook. [file 13223_2024_933_MOESM1_ESM.pdf]

HOW TO MANAGE FOOD PROTEIN-INDUCED ALLERGIC PROCTOCOLITIS (FPIAP)

What is FPIAP?

It is a common cause of blood-streaked stool in otherwise well-appearing infants, and typically begins within the first few months of life. It is characterized by an immune response that results from exposure to a food allergen through breast milk or formula. This can result in inflammation of your baby's developing gastrointestinal tract (GI), resulting in blood and mucus in baby's stool.

Treatment involves elimination of the culprit food from the baby's diet, through either elimination of the food from the breastfeeding parent's diet (breastfed infants) or a change in formula (formula fed infants).

Dietary Triggers

The most common culprit foods are: cow's dairy (70-80%), egg, soy and corn. Some children may react to more than one food.

Many breastfed infants that do not respond to this diet do not respond to further food restrictions, and elimination diets can cause harm in breastfeeding parents. Your care provider may recommend transition to a hypoallergenic formula.

About 90% of patients tolerate soy formula ($) and ~98% tolerate extensively hydrolyzed cows milk formula ($$). Amino acid formula ($$$) can be used for those that still have symptoms.

What does FPIAP look like? Blood and / or mucus in the diaper

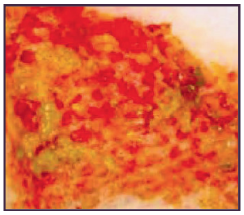
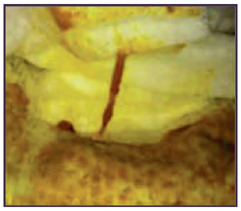
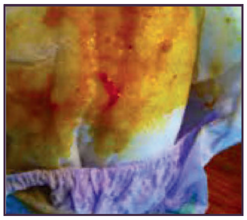
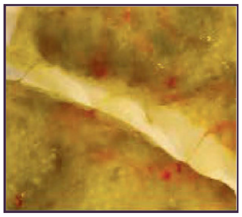

---

1 Find Culprit Food via Elimination Strategy

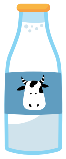
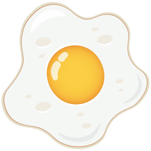
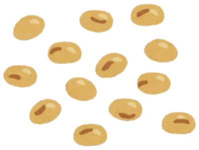
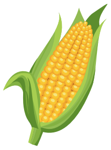

A **Remove Dairy 5 days** → Blood still in stool → B **Remove Egg 5 days** → Blood still in stool → C **Remove Soy 5 days** → Blood still in stool → D **Remove Corn 5 days**

- If blood persists for 1 week, or mucus persists for 1 month after removal of all foods, discuss with your healthcare provider.
- Once symptoms resolve, stop further elimination, proceed to Part 2, and reintroduce eliminated food(s) in reverse order.

Re-introduce Foods Once Stool Free of Blood and Mucus 2

Add food back into diet, in reverse order, either directly (in an age appropriate form), or via breast milk. Symptoms typically appear within 5 days.

E **Add Corn 5 days** → No blood in stool → F **Add Soy 5 days** → No blood in stool → G **Add Egg 5 days** → No blood in stool → H **Add Dairy 5 days**

- If blood in stool restarts after adding back one culprit food, avoid this food for the next 6 to 9 months.

FREQUENTLY ASKED QUESTIONS

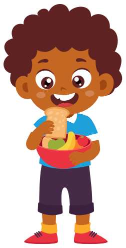

Will my child be able to eat the food that caused them to have FPIAP?

Most children outgrow FPIAP by 6 to 14 months of age. Once your child is 9 months old, the food can be reintroduced. If the mucous and blood do not return, the disease has resolved. The food can be incorporated into the diet of the child and the breastfeeding parent. Children with FPIAP are not at increased risk of any other type of allergic reaction to this food, or allergies to other foods. There are also no increased risk of other diseases such as inflammatory bowel disease in infants with this condition. There is a small risk of anemia if symptoms are severe and untreated. For all infants, initial doods offered to infants should be iron rich.

Are vitamins or supplements needed?

Exclusively or partially breastfed babies should take a daily Vitamin D supplement of 400 IU. Breastfeeding parents should get 1000 mg calcium and 600 IU Vitamin D daily. If you are breastfeeding and avoiding multiple foods, consider speaking to a dietitian.

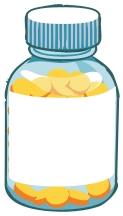

The following resources may be helpful:
1. https://osteoporosis.ca/calcium-calculator/
2. https://www.unlockfood.ca/en/Articles/Vitamins-and-Minerals/What-You-Need-to-Know-about-Calcium.aspx

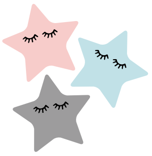

My baby isn't sleeping well. Is it a food allergy?

The main cause of sleep issues in children this age is colic and teething. Occasionally children with FPIAP have improved sleep with colic symptoms when their trigger food is eliminated.

When can I start solids?

Infants are developmentally ready for solids when they:

- Are close to 6 months of age, and birth weight has doubled.
- Can sit by themselves, alone or with support.
- Can watch a spoon and open their mouth when they see it coming.
- Turn their head away when they do not want something
- Close lips over a spoon and keep at least some of the food in their moth, rather than pushing it back out with their tongue.

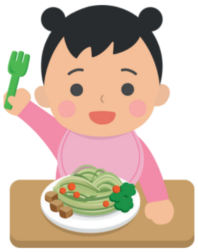

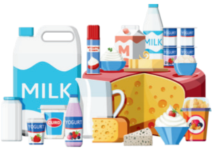

Dairy elimination helped, but things suddenly got bad again. Why?

This is usually the result of accidental exposure to diary, which can appear in unexpected places! Non vegan margarine is a common culprit for accidental exposures. If you are unsure, talk to your Registered Dietitian or Allergist; they may be able to assist removing "hidden" sources of FPIAP causing proteins.

When should I seek urgent medical advice?

Most Infants with FPIAP are otherwise healthy and growing well. The following symptoms are not caused by FPIAP, and would be a reason to see your health care provider urgently:

- Difficulties gaining weight or weight loss
- Signs of dehydration (reduced urination)
- Lethargy (unusually tired, floppy or difficult to rouse)

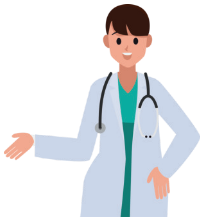

References:
1. Lake AM Food induced eosinophilic proctocolitis. J Pediatr Gastroenterol Nutr 2000; 30 Suppl S58-60.
2. Nowak-Wegrzyn A. Food protein-induced enterocolitis syndrome and allergic proctocolitis. Allergy Asthma Proc 2015; 36:172-187; doi 10.2500/aap.2015.36.3811

Handout generated by: Francesca Reinbolt, Delane Peters & Scott Cameron
Updated by: Matt Griffin, Victoria Cook & Scott Cameron, October 2024
